# Evaluation of an oral health program for children in San Francisco de Macorís, Dominican Republic (2019–2024)

**DOI:** 10.3389/froh.2025.1671953

**Published:** 2025-12-09

**Authors:** David Ribas-Perez, Carlos Muñoz-Viveros, Angel Luis Formoso-Veloso, Francisco Jesus Carrillo-Sanchez, Antonio Castaño-Seiquer

**Affiliations:** 1 Department of Stomatology, University of Seville, Seville, Spain; 2 Kerr Corporation, Orange, CA, United States

**Keywords:** oral health, oral health-related quality of life, Dominican republic, DMFT, COHIP19 SF

## Abstract

**Introduction:**

Oral diseases remain a public health concern in the Dominican Republic, with epidemiological data indicating greater severity compared to other countries with similar geo-economic profiles. Numerous organizations, both governmental and private, have been involved in addressing this issue. Various nonprofit organizations have implemented oral health initiatives in the form of programs aimed at mitigating this situation. However, these projects often lack validation through studies assessing their impact on oral health outcomes.

**Objective:**

The objective of this study is to describe the oral health status of a pediatric population and its association with perceived quality of life, while also evaluating the impact of a specific oral health program conducted in the city of San Francisco de Macorís. The evaluation aims to identify areas for improvement in the program's design and implementation.

**Methods:**

The oral health program was assessed over the period 2019–2024. Oral health status was measured using a World Health Organization (WHO)-based survey, and oral health-related quality of life was assessed with the culturally adapted Spanish version of the COHIP-19SF questionnaire. The impact of the program was analyzed using various health and quality-of-life indicators.

**Results:**

At baseline in 2019, 94 children from three regions in the Dominican Republic were assessed. Over five years, the restoration index improved substantially from 31.4% to 86.2%, indicating better access to dental care. Quality of life, measured using the COHIP-SF19, also improved. Significant gains were seen in functional well-being, oral health, and self-image domains, while socio-emotional well-being remained unchanged. Overall, the total COHIP-SF19 score dropped by 4 points, reflecting a meaningful improvement in oral health-related quality of life.

**Conclusion:**

The intervention significantly reduced primary tooth decay, improved treatment access, and enhanced children's oral health-related quality of life. These results support the value of sustained oral health programs in vulnerable communities.

## 1 Introduction

Oral diseases—particularly dental caries—continue to represent one of the leading global public health concerns, affecting a significant proportion of the population, especially children and adolescents, and predominantly in underserved communities (1, 2).

In the case of the Dominican Republic, various studies have reported alarmingly high rates of dental caries prevalence. One study conducted in Santo Domingo revealed a caries prevalence of 90.02%, what means that only 9.98% of adolescents were found to be caries-free. These epidemiological indicators are considerably higher than those reported in neighboring countries within the same geographic region (3).

Data from the World Health Organization's Oral Health Program, CAPP (Country/Area Profile Programme), provide comparative prevalence figures for oral diseases. This database allows for benchmarking among countries, and it reveals that the Dominican Republic shows significantly higher prevalence rates of dental caries and mean DMFT scores compared to other Caribbean nations (4–17) (Table 1).

**Table 1:** DMFT Index at Age 12 in Caribbean and central American countries.

Country	Year	Age	Prevalence (%)	DMFT (12 years)
Antigua and Barbuda (5)	2,006	12	35.9	0.9
Bahamas (the) (6)	2005	11	19.39	1.56
Barbados (7)	2001	12	37	0.86
Bermuda (8)	2009	12		0.54
Costa Rica (5)	2006	12	72	2.5
Cuba (5)	2005	12		1.5
Dominica	2006	12		1.2
Dominican Republic (the) (9)	2008	12	73	2.64
El Salvador (10)	2008	12		1.45
Grenada (5)	2000	12		2.2
Guatemala (4)	2002	12		4.5
Haiti (11)	1999	12	31	0.65
Honduras (12)	1997	12	83.4	3.7
Jamaica (13)	1995	12	40.9	1.1
Nicaragua (14)	2002	12	45	1.5
Panama (5)	2008	12		3.6
Puerto Rico (15)	2010	12	69	2.5
Saint Lucia (16)	2005	12		3
Suriname (16)	2002	12		1.9
Trinidad and Tobago (17)	2004	12	34	0.6

*Source:* WHO Oral Health—CAPP, Malmö University (4).

Previous studies conducted by our research group in public schools in the Dominican Republic served as the foundation for the development of public health programs targeting this population. These studies indicated that approximately 70% of 12-year-old schoolchildren presented with active dental caries, reflecting a high burden of disease in this population (18). Similarly, research carried out in 26 schools across the country revealed an overall prevalence of dental caries in that study was 90.02% (19).

Several public and private initiatives have been developed to address this public health issue. One notable example is the “Caries-Free School” program led by the Dominican National Health Service (Servicio Nacional de Salud, SNS), which serves over 130,000 children across 250 public schools, promoting good oral hygiene habits from an early age. Additionally, the SNS provides oral healthcare to more than one million Dominicans through 150 primary care centers. Educational programs such as “Bright Smiles, Bright Futures” have also reached thousands of children and their families, emphasizing oral health education and dental caries prevention (20).

Nonetheless, chronic challenges persist—particularly limited access to dental care services. Despite progress, the unequal distribution of dentists and lack of resources in rural areas perpetuate disparities in oral healthcare access. Coverage remains insufficient, especially in rural and marginalized communities. Inadequate infrastructure, including the absence of clean water and appropriate facilities in schools, further undermines the effectiveness of interventions, thereby negatively impacting both oral health and quality of life.

Given this context, the involvement of private initiatives and NGOs—such as Envista Corporation (EnvistaCo) and the Luis Séiquer Foundation for Social Dentistry—is essential. These organizations provide free dental care in underserved communities, with a strong focus on prevention and oral health education.

The present study was undertaken to assess the impact of an oral health program implemented since 2019 in a specific area of San Francisco de Macorís. Its aim is to determine whether the program is truly benefiting the population—not only through individual case resolution, where its utility is clear, but also in terms of broader public health outcomes.

Oral health is fundamental to a healthy and fulfilling life. It is therefore crucial that all sectors work collaboratively to improve oral health-related quality of life in the Dominican Republic.

## 2 Methods

### 2.1 Study type, settings and intervention details of the program

Over a five-year period (2019–2024), an oral health program was implemented for a defined pediatric population in the city of San Francisco de Macorís, Dominican Republic—specifically, in the Vista del Valle neighborhood. Initially, 94 children aged between 4 and 16 years were included in the study. These children attended the Dental Outreach Program, co-organized by professionals from private dental practice (USA), the University of Seville (US, Spain), and the Universidad Católica Nordestana (UCNE, Dominican Republic), and were randomly selected in 2019.

Subsequently, the program continued to be implemented every six months until 2024 (except for 2020 due to COVID-19 restrictions), providing free oral health care services to the community.

The program involved dental examinations for all schoolchildren to determine their treatment needs. Every child received standardized oral hygiene instruction and a kit containing preventive materials (toothbrushes and fluoride toothpaste at 1,450 ppm). Based on individual needs, children received any necessary dental treatment. The treatments provided encompassed preventive measures, including fissure sealants and fluoride varnish applications, restorative procedures such as fillings, and extractions when clinically indicated. Pulp treatments were not included due to the absence of radiographic assessments, which limited the ability to accurately diagnose pulp pathology.

For the initial selection sample, all children whose parents or legal guardians provided consent and who did not present any severe systemic condition that could bias the outcomes were included in the study. Written informed consent was obtained at the beginning of the program, following the ethical principles outlined in the Declaration of Helsinki (Edinburgh amendment, 2000). The study was approved by the Ethics Committee of the Fundación Odontología Social (protocol code 002/19, approved July 15, 2019).

### 2.2 Data collection

As in 2019, data collection was conducted again in 2024, in two stages. First, clinical oral examinations were performed using portable dental units to assess the children's oral health, following the methodology recommended by the World Health Organization in its publication *Oral Health Surveys*: Basic Methods, 5th edition (21).

Afterward, parents completed the COHIP-19SF questionnaire, which has been validated for use in Spanish-speaking populations. This questionnaire was designed to evaluate the perception of Oral Health Related Quality of Life (OHRQoL) in children and adolescents. It was developed, encompassing four key dimensions: oral function, functional well-being, socioemotional well-being and self-image. COHIP-SF19 scores were calculated according to standard conventions, with lower scores indicating better oral health-related quality of life (OHRQOL).

The instrument comprised 19 items, each rated on a 5-point Likert scale ranging from “Never” to “Always”, allowing for the assessment of the frequency or intensity of oral health-related issues and their impact on the participants' lives.

Prior to its full application, the questionnaire underwent a pilot study involving face validity assessment by two independent examiners, who reviewed the instrument for clarity, relevance, and comprehensibility. This preliminary test also served to evaluate the internal consistency and alignment of the results with theoretical expectations. All clinical assessments throughout the program were conducted by the same team of examiners. To ensure consistency and reliability, inter-examiner reliability tests were performed prior to the final evaluation in the fifth year (kappa inter-examiner index = 0.88.) Examiners were trained and calibrated at the beginning of the program and periodically reassessed to maintain standardization.

### 2.3 Study variables and statistical analysis

Statistical analyses were performed using R statistical software (version 4.5). Descriptive statistics for baseline characteristics were presented as means with standard deviations (SD) for continuous variables and frequencies with percentages for categorical variables. To evaluate changes between 2019 and 2024, multilevel Poisson regression models with random intercepts for participants nested within regions were fitted.

The primary outcomes analyzed included primary tooth decay (dft), permanent tooth decay (DMFT), and the restoration index. Models were adjusted for potential confounders, specifically age, sex, Dean Modified Fluorosis Index, and malocclusion. Estimated marginal means (EMMs) were calculated and compared between years, with Dunnett adjustments applied for multiple comparisons to control the family-wise error rate.

Results were expressed as regression coefficients (*β*) or adjusted mean differences, with corresponding 95% confidence intervals (CI) and *p*-values. A *p*-value <0.05 was considered statistically significant. Similar multilevel Poisson models, incorporating year as covariate, were employed to analyze domain-specific scores of the COHIP-SF19 scale, assessing changes in Functional Well-Being, Oral Health, Self-Image, and Socio-Emotional Well-Being.

## 3 Results

### 3.1 Participant baseline characteristics and follow up

**Figure 1:**
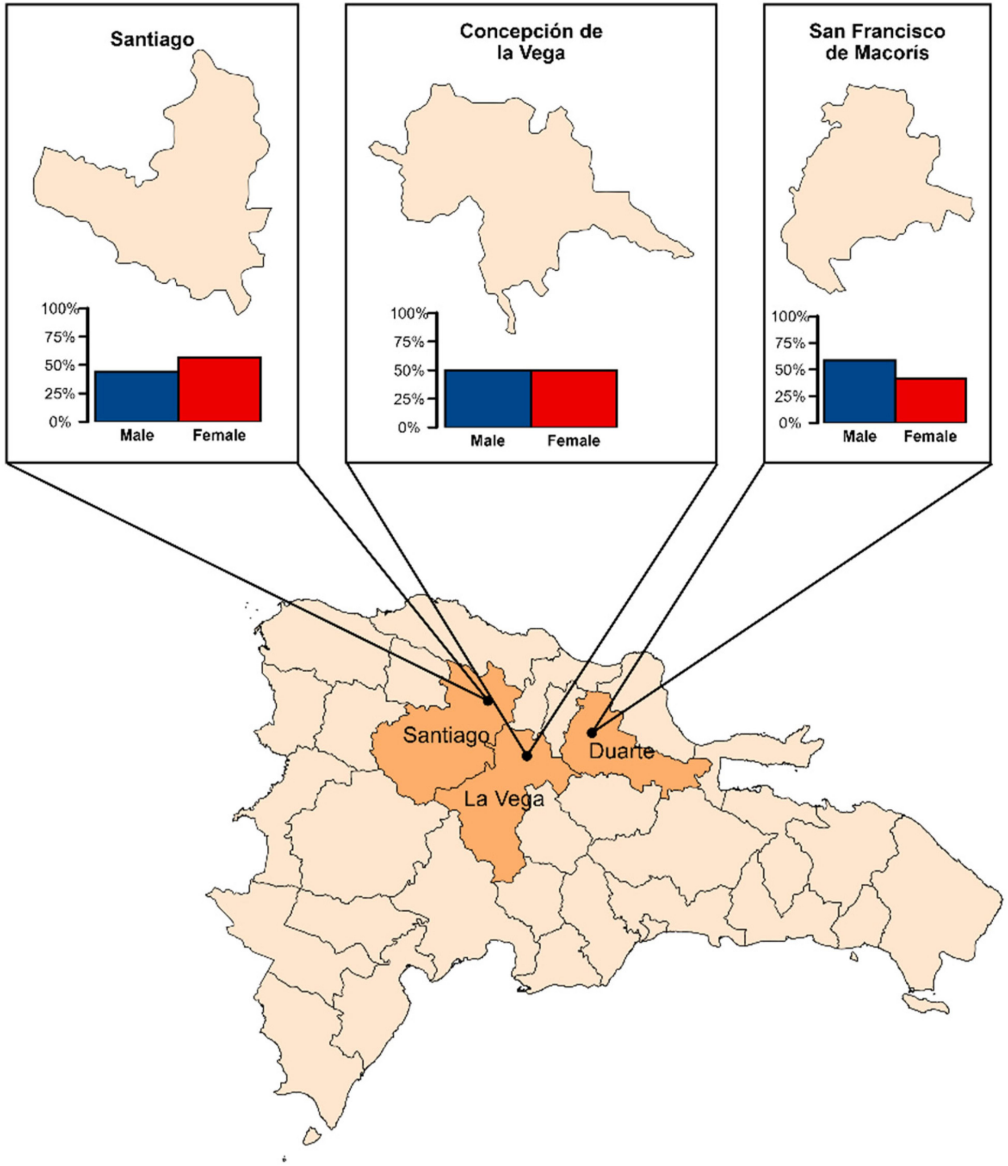
Study municipalities in the Dominican republic and sex distribution of enrolled children by site.

Of the 94 children initially enrolled in the program in 2019, 70 were from San Francisco de Macorís (mean age 10.0 ± 3.1 years; 41 males, 29 females), 16 from Santiago (mean age 11.8 ± 3.8 years; 7 males, 9 females) and 8 from Concepción de la Vega (mean age 10.9 ± 4.8 years; 4 males, 4 females) (Figure 1). At baseline, most participants exhibited no fluorosis [San Francisco de Macorís 62/70 [88.6%], Santiago 13/16 [81.3%], Concepción de la Vega 7/8 [87.5%]], while mild fluorosis (index = 1) affected 5/70 (7.1%), 1/16 (6.3%) and 1/8 (12.5%), moderate fluorosis (index = 2) was seen in 2/70 (2.9%) and 2/16 (12.5%), and severe fluorosis (index = 3) occurred in 1/70 (1.4%), all in San Francisco de Macorís. Malocclusion was absent in 32/70 (45.7%), 5/16 (31.3%) and 5/8 (62.5%); mild in 35/70 (50.0%), 10/16 (62.5%) and 3/8 (37.5%); and moderate to severe in 3/70 (4.3%) and 1/16 (6.3%), with no cases in Concepción de la Vega.

Of the 94 children initially enrolled, not all completed the study due to changes in residence, which necessitated school transfers, or dropout, particularly among those nearing the end of compulsory schooling. In any five-year longitudinal study, some attrition is expected and may influence the results. Our initial sample size calculations anticipated about a 20% dropout rate, and by the conclusion of the study in 2024, a total of 70 children had completed the program, thus aligning with our predefined expectations.

Regarding the DMFT index, the overall mean score for the sample in 2024 was 1.86 (SD = 1.56), with a mean dft component of 0.42 (SD = 1.01). The values of DMFT are similar to those recorded in 2019 (see Table 2). However, the Restoration Index showed a marked improvement, increasing to 82% compared to 26% in 2019, indicating a significant reduction in unmet treatment needs as a result of the oral health program.

**Table 2:** Total dft, DMFT and restoration Index (RI) of the sample (2019 and 2024). Mean and standard deviation (SD) or percetage.

	2019				2024			
	Decayed	Missing	Filled	Total	Decayed	Missing	Filled	Total
dft	1.49	–	0.37	1.86 (2.04)	0.08	–	0.34	0.42 (1.01)
DMFT	1.10	0.08	0.52	1.70 (1.90)	0.22	0.12	1.52	1.86 (1.56)
RI (%)	26%				82%			

### 3.2 Impact of the oral health program on dft, DMFT and restoration Index

In multilevel Poisson regression analyses adjusted for age, sex, fluorosis, and malocclusion, primary tooth decay (dft) counts significantly decreased from 2019 to 2024 (β = –1.588; 95% CI −1.980 to −1.196; *p* < 0.001) (Supplementary Table S1).

**Figure 2:**
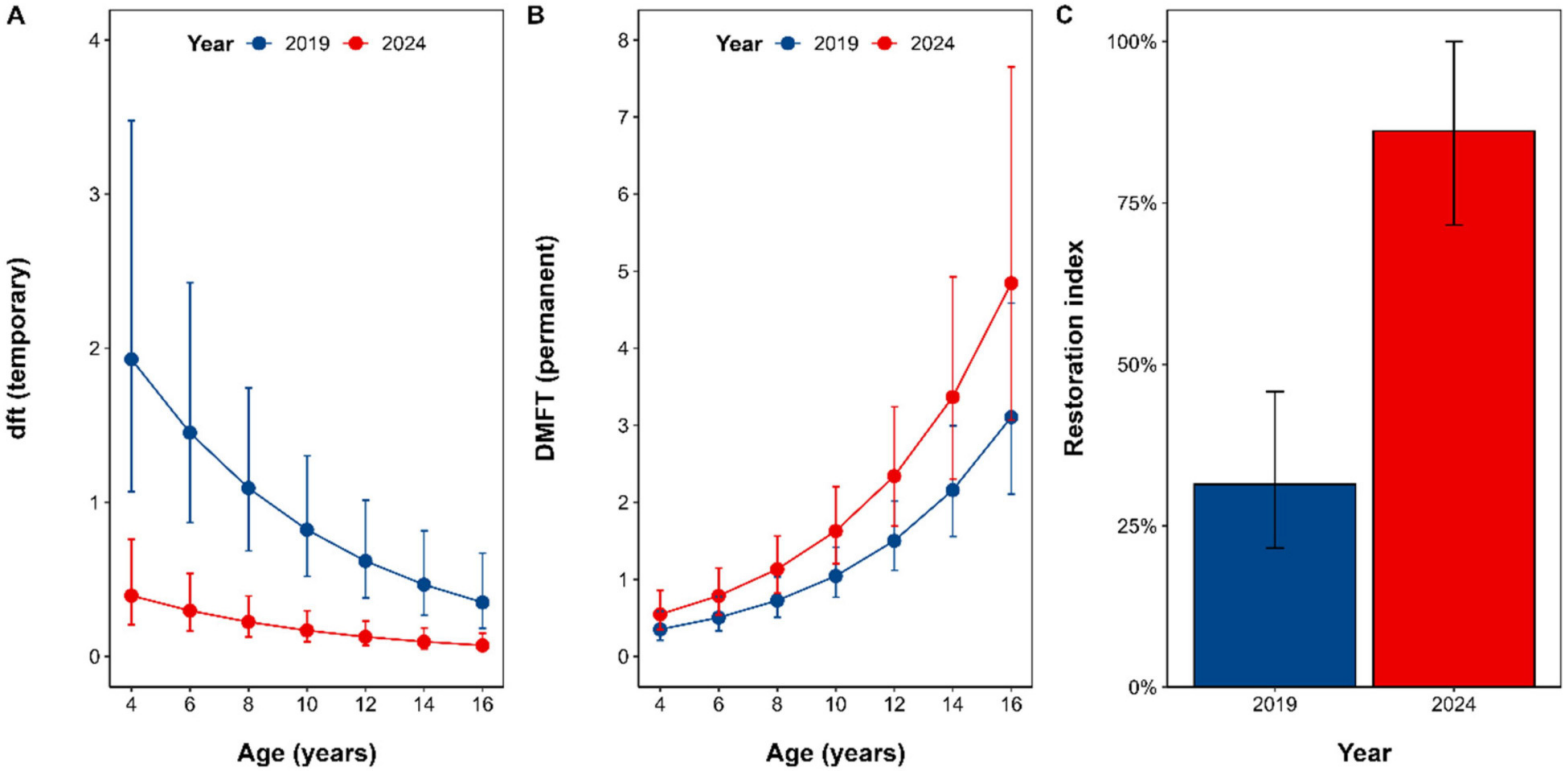
Temporal trends in dft, DMFT, and restoration Index by Age (2019 vs. 2024). Data are presented as model-estimated means (±95% CI) for primary decay **(dft; Panel A)**, permanent decay **(DMFT; Panel B)** and restoration index **(Panel C)** in 2019 (blue) and 2024 (red). Estimates derive from multilevel Poisson regressions with random intercepts for participants nested within region, adjusted for age, sex, Dean modified fluorosis index and malocclusion. All age-specific comparisons employ Dunnett`s adjustments for multiple testing.

The greatest differences were observed at younger ages, particularly at age 4 (estimate = −1.534; 95% CI −2.797 to −0.272; *p* = 0.009), with a progressive reduction in magnitude as age increased. Differences remained statistically significant at all ages evaluated (ages 6, 8, 10, 12, 14, and 16) (Figure 2A) (Supplementary Table S2).

Conversely, permanent tooth decay (DMFT) significantly increased overall between 2019 and 2024 (β = 0.445; 95% CI 0.178–0.711; *p* = 0.001) (Supplementary Table S3).

Age-specific comparisons indicated statistically significant differences from ages 4–12, with the largest increase observed at age 12 (estimate = 0.840; 95% CI 0.050–1.631; *p* = 0.0313). However, at ages 14 and 16, increases were not statistically significant after adjustment for multiple comparisons (Figure 2B) (Supplementary Table S4).

Finally, restoration rates increased from 31.4% (95% CI 21.5–45.8%) in 2019 to 86.2% (95% CI 71.6%–100%) in 2024, representing a 54.8–percentage-point gain (95% CI 34.9–74.6; *p* < 0.001) (Figure 2C).

### 3.3 Changes in oral health-related quality of life (COHIP-SF19) domains

**Figure 3:**
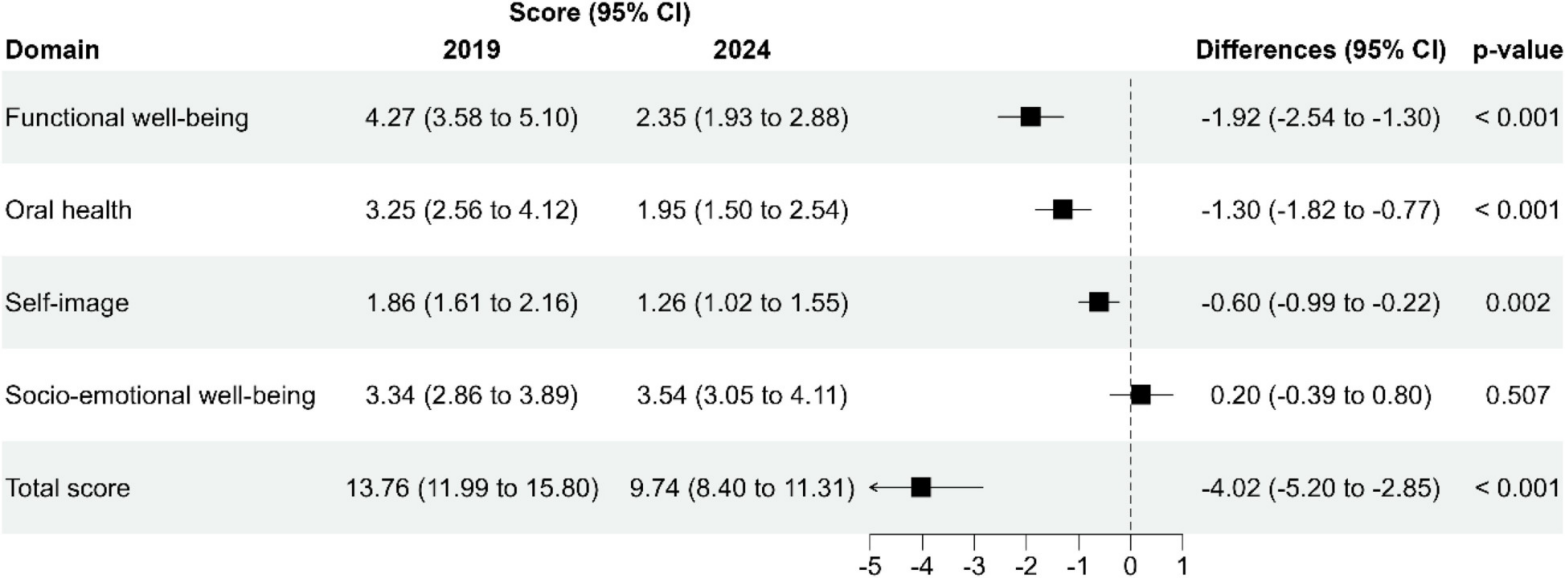
Domain-specific changes in COHIP-SF19 scores between 2019 and 2024.

Figure 3 presents the domain-specific COHIP-SF19 scores in 2019 and 2024 along with the mean differences (±95% CI) derived from multilevel Poisson regression models (random intercepts for participants nested within regions), adjusting for year. Significant improvements (reductions in scores) were observed between 2019 and 2024 for Functional Well-Being (difference: −1.92; 95% CI −2.54 to −1.30; *p* < 0.001), Oral Health (−1.30; 95% CI −1.82 to −0.77; *p* < 0.001), and Self-Image (−0.60; 95% CI −0.99 to −0.22; *p* = 0.002). Socio-Emotional Well-Being did not show significant changes over time (difference: 0.20; 95% CI −0.39–0.80; *p* = 0.507). The total COHIP-SF19 score significantly improved, decreasing from 13.76 (95% CI 11.99–15.80) in 2019–9.74 (95% CI 8.40–11.31) in 2024, representing a mean difference of −4.02 (95% CI −5.20 to −2.85; *p* < 0.001).

## 4 Discussion

Results obtained in this study demonstrate a significant improvement in oral health and oral health-related quality of life among children participating in the oral health program implemented in San Francisco de Macorís between 2019 and 2024. The reduction in caries prevalence and in the DMFT and dft indices, along with improvements in COHIP-19SF dimensions, reflects the positive impact of the intervention.

### 4.1 Caries prevalence and program efficacy

The initial caries prevalence in the 2019 sample was 80.9%, with a DMFT index of 1.70 (1.90 SD) and a dft index of 1.86 (2.04 SD), figures consistent with those reported in previous studies conducted in the Dominican Republic (18). After five years of intervention, a significant reduction in caries prevalence was observed, decreasing from 80.9% to 26% in 2024. Although the DMFT index in 2024 reached 1.86 (1.45 SD), suggesting a potential increase, this trend was not reflected in the dft index, which remained low at 0.42 (1.01 SD). It is important to consider that the sample population was aged by five years during the study period, which naturally influences DMFT/dft values due to the transition from primary to permanent dentition. More importantly, the untreated caries component (D/d) showed a marked decrease, indicating improved access to care and treatment uptake.

These findings suggest the program's effectiveness in the prevention and treatment of dental caries [the filled component (F) of the DMFT index also increased, reaching a restoration index of 82%]. A primary consideration concerns the index used for comparison (DMFT/d). At first glance, the increase in the index in the studied population (from 1.70 to 1.86) might suggest no program impact; however, a critical analysis of this index, as described by Fraihat et al. in 2019 (22), recommends examining the restoration component to evaluate dental service effectiveness in a population. Indeed, the restoration index (percentage of filled teeth relative to total DMFT) increased from 26% to 82% (18).

As indicated in the introduction, there is a significant lack of epidemiological data in the Dominican Republic, although existing reports show very high caries indices, especially untreated caries, exceeding those of other Caribbean countries.

These data contrast with those from developed countries (USA and Europe), where oral health indicators are considerably better, supported by epidemiological monitoring of oral diseases conducted approximately every five years. Examples include Germany, Sweden, and Spain, with DMFT scores at 12 years of 0.5 in 2014, 0.65 in 2021, and 0.58 in 2021, respectively (4, 23–25).

Public health experts debate the long-term benefits of purely preventive or oral health promotion programs vs. intervention-focused programs. While preventive programs are more cost-effective (22), we advocate for addressing urgent oral health problems promptly. Managing pain and infection should take priority in critical situations. This does not imply neglecting prevention and health promotion, which must remain core components of any program.

Over these five years, our program began with a situational analysis which revealed an evident need for treatment, prioritizing caries and oral infection management. Alongside, we implemented a more ambitious subprogram targeting the entire population studied in San Francisco de Macorís for specific caries prevention, with essential local community involvement (health promoters, schoolteachers), and distribution of fluoridated toothpaste (1,450 ppm sodium fluoride) and toothbrushes to all examined individuals. Similar approaches have been successfully implemented elsewhere (26–30).

These results align with those from other regional oral health programs, such as the initiative by Fundación Bocas Sanas Holanda-Maimón in Puerto Plata, which also reported improvements in caries indices following intervention (31). It is worth highlighting that the most significant finding directly attributable to the preventive intervention is the decreasing trend observed in the dft index (Figure 2A). A reduction in dft within an aging pediatric cohort serves as a strong indicator of the effectiveness of the implemented preventive measures, as it reflects a lower incidence of caries during the critical stage of primary dentition.

### 4.2 Impact on oral health-related quality of life

Application of the COHIP-19SF questionnaire revealed significant improvements (reductions in scores) for Functional Well-Being, Oral Health, and Self-Image. Socio-Emotional Well-Being did not show significant changes over time. The total COHIP-SF19 score significantly improved, decreasing from 13.76 (95% CI 11.99–15.80) in 2019–9.74 (95% CI 8.40–11.31) in 2024, representing a mean difference of −4.02 (95% CI −5.20 to −2.85; *p* < 0.001) suggesting that although the program positively impacted other aspects of quality of life, perceptions of socio-emotional well-being may require additional interventions. These findings may suggest that such perceptions could not be directly responsive to interventions limited to dental care alone. It may be more effective to consider programs that adopt a holistic approach to oral health, integrating psychological and socioeconomic factors, in order to more comprehensively address the determinants of socio-emotional well-being in the population (32, 33).

Tus is also consistent with studies in other pediatric populations where community-based interventions improved oral health-related quality of life (35) Bramantoro et al., in their systematic review, reported that school-based oral health promotion programs improved OHRQoL, especially when involving children, teachers, and parents. Similarly, Rochmah et al. highlighted that programs engaging the study population have greater cost-effectiveness benefits (34, 35).

Our oral health program has been continuously supported by local institutions. The Universidad Católica Nordestana (UCNE) provided logistical and human resource support throughout, while the school hosting the program facilitated its implementation. We attribute the positive results largely to the commitment of a community with a recognized need for dental healthcare.

### 4.3 Socioeconomic considerations and program sustainability

Despite positive outcomes, challenges remain concerning access to dental services, especially in rural and marginalized communities. The unequal distribution of dentists and lack of resources in these areas complicate the sustainability of programs like the one implemented in San Francisco de Macorís. Partnerships with universities such as UCNE, which offers dentistry education, may help address these issues.

Previous studies have emphasized the importance of addressing social determinants of health in oral health program planning to ensure efficacy and sustainability, as supported by Tahani et al. (36).

A limitation of our study is that, although important determinants such as age, sex, fluorosis, and malocclusion were controlled for, other relevant factors, including socioeconomic status, diet, and parental education, were not included. This omission was due to the lack of availability of these data in the database used and constraints inherent to the original study design. We acknowledge that these factors may have a significant impact on the observed outcomes, and their exclusion limits the generalizability of our findings. Future studies should consider these determinants to gain a more comprehensive understanding of the factors influencing child oral health.

## 5 Conclusion

The oral health program implemented in San Francisco de Macorís has proven effective in reducing the prevalence of dental caries and improving the oral health-related quality of life of participating children. However, it is essential to continue implementing strategies that address the social determinants of health and ensure the sustainability of these interventions to achieve a lasting impact on children's oral health in the Dominican Republic.

Based on the analysis of existing gaps, we recommend the implementation of the following measures:

**Strengthen oral health education:** Implement continuous educational programs in schools and communities to promote oral hygiene and prevent oral diseases.
**Expand dental service coverage:** Increase the dental care network in rural and marginalized communities, ensuring equitable access to quality services.
**Reduce dental treatment costs:** Promote policies that make dental treatments more economically accessible to the entire population.
**Encourage public-private collaboration:** Establish partnerships among government, non-governmental organizations, and the private sector to implement oral health prevention and treatment programs.

## Data Availability

The raw data supporting the conclusions of this article will be made available by the authors, without undue reservation.
