# Supplementary material for: Evaluation of an oral health program for children in San Francisco de Macorís, Dominican Republic (2019–2024)
Source: Front Oral Health. 2025 Dec 9;6:1671953. doi: 10.3389/froh.2025.1671953 (PMC12722982; doi:10.3389/froh.2025.1671953)
Supplement: Supplementary file 1 [file Table1.docx]

# Supplementary material

**Table S1.** Multilevel Poisson regression results for temporary decay (dft) adjusting for age, sex, fluorosis and malocclusion

| **Characteristic** | **β** | **95% CI** | **p-value** |
| --- | --- | --- | --- |
| Year |  |  |  |
| 2019 | — | — |  |
| 2024 | -1.588 | -1.980, -1.196 | <0.001 |
| Age – 10, years | -0.142 | -0.211, -0.073 | <0.001 |
| Sex |  |  |  |
| Male | — | — |  |
| Female | 0.355 | -0.107, 0.817 | 0.133 |
| The Dean Modified Fluorosis Index |  |  |  |
| 0 | — | — |  |
| ≥ 1 | -1.096 | -1.957, -0.236 | 0.013 |
| Malocclusion |  |  |  |
| No malocclusion | — | — |  |
| Malocclusion | -0.233 | -0.699, 0.233 | 0.328 |
| Abbreviation: CI, confidence interval | | | |

**Table S2.** Age-specific adjusted differences in dft between 2024 and 2019

| **Age, years** | **2019** | **2024** | **Differences (95% CI)^¶^** | **p-value** |
| --- | --- | --- | --- | --- |
| 4 | 1.93 (1.07 - 3.48) | 0.39 (0.20 - 0.76) | -1.53 (-2.80 to -0.27) | 0.009 |
| 6 | 1.45 (0.87 - 2.43) | 0.30 (0.16 - 0.54) | -1.15 (-1.99 to -0.32) | 0.002 |
| 8 | 1.09 (0.68 - 1.74) | 0.22 (0.13 - 0.39) | -0.87 (-1.44 to -0.29) | < 0.001 |
| 10 | 0.82 (0.52 - 1.30) | 0.17 (0.10 - 0.30) | -0.65 (-1.08 to -0.23) | < 0.001 |
| 12 | 0.62 (0.38 - 1.01) | 0.13 (0.07 - 0.23) | -0.49 (-0.83 to -0.15) | < 0.001 |
| 14 | 0.47 (0.27 - 0.81) | 0.10 (0.05 - 0.18) | -0.37 (0.66 to -0.09) | 0.004 |
| 16 | 0.35 (0.18 - 0.67) | 0.07 (0.03 - 0.15) | -0.28 (-0.53 to -0.03) | 0.018 |
| ^¶^ Averaged over the levels of: Sex, Dean’s Modified Fluorosis Index and Malocclusion. Confidence levels were adjusted using the Dunnett’s method for seven estimates, and p-values were adjusted using the same method for seven tests.  Abbreviation: CI, confidence interval. | | | | |

**Table S3.** Multilevel Poisson regression results for permanent decay (DMFT) adjusting for age, sex, fluorosis and malocclusion

| **Characteristic** | **β** | **95% CI** | **p-value** |
| --- | --- | --- | --- |
| Year |  |  |  |
| 2019 | — | — |  |
| 2024 | 0.445 | 0.178, 0.711 | 0.001 |
| Age – 10, years | 0.182 | 0.126, 0.238 | <0.001 |
| Sex |  |  |  |
| Male | — | — |  |
| Female | 0.219 | -0.114, 0.552 | 0.197 |
| The Dean Modified Fluorosis Index |  |  |  |
| 0 | — | — |  |
| ≥ 1 | -0.331 | -0.838, 0.176 | 0.200 |
| Malocclusion |  |  |  |
| No malocclusion | — | — |  |
| Malocclusion | 0.027 | -0.314, 0.368 | 0.878 |
| Abbreviation: CI, confidence interval | | | |

**Table S4.** Age-specific adjusted differences in DMFT between 2024 and 2019

| **Age, years** | **2019** | **2024** | **Differences (95% CI)^¶^** | **p-value** |
| --- | --- | --- | --- | --- |
| 4 | 0.35 (0.21 - 0.59) | 0.55 (0.35 - 0.86) | 0.20 (0.02 to 0.37) | 0.022 |
| 6 | 0.50 (0.33 - 0.77) | 0.79 (0.54 - 1.15) | 0.28 (0.04 to 0.53) | 0.016 |
| 8 | 0.72 (0.51 - 1.03) | 1.13 (0.82 - 1.56) | 0.41 (0.05 to 0.76) | 0.016 |
| 10 | 1.04 (0.77 - 1.42) | 1.63 (1.20 - 2.20) | 0.58 (0.06 to 1.11) | 0.020 |
| 12 | 1.50 (1.12 - 2.02) | 2.34 (1.69 - 3.24) | 0.84 (0.05 to 1.63) | 0.031 |
| 14 | 2.16 (1.56 - 2.99) | 3.37 (2.30 - 4.93) | 1.21 (-0.01 to 2.43) | 0.053 |
| 16 | 3.11 (2.10 - 4.58) | 4.84 (3.07 - 7.65) | 1.74 (-0.16 to 3.64) | 0.090 |
| ¶ Averaged over the levels of: Sex, Dean’s Modified Fluorosis Index and Malocclusion. Confidence levels were adjusted using the Dunnett’s method for seven estimates, and p-values were adjusted using the same method for seven tests.  Abbreviation: CI, confidence interval. | | | | |
